# Spectrophotometric Estimation of Polyphenolic Compounds in Willowherbs (*Epilobium angustifolium* L. and *E. hirsutum* L.) and Implications for Genetic Resource Conservation

**DOI:** 10.3390/plants15060911

**Published:** 2026-03-16

**Authors:** Juozas Labokas, Akvilė Vilutytė

**Affiliations:** 1 Laboratory of Economic Botany, State Scientific Research Institute Nature Research Centre, Akademijos g. 2, LT-08412 Vilnius, Lithuania; 2 Pharmacy and Pharmacology Center, Institute of Biomedical Sciences, Faculty of Medicine, Vilnius University, Geležinio Vilko g. 29A, LT-01112 Vilnius, Lithuania

**Keywords:** *Epilobium*, extract, flavonoids, flowers, leaves, phenolic compounds, population

## Abstract

There is a growing interest in natural bioactive substances, particularly plant-derived secondary metabolites. Polyphenols constitute one of the largest and most significant groups of these metabolites. Rosebay willowherb (*Epilobium angustifolium*) is well known in traditional medicine and can serve as a reference species for studying its less-known congener, hairy willowherb (*E. hirsutum*), thereby expanding knowledge of medicinal plants. This study aimed to quantitatively estimate and compare the total phenolic content (TPC) and total flavonoid content (TFC) in the leaves and flowers of *Epilobium angustifolium* and *E. hirsutum*, and to identify populations with the highest phytochemical potential. TPC and TFC were quantified using the Folin–Ciocalteu and aluminium chloride (AlCl_3_) colorimetric assays, respectively, with resulting values regarded as estimates due to the non-specificity of these assays. The results showed that, in terms of TPC, *E. angustifolium* leaves accumulated 132 ± 3.4 mg_GAE/g (milligrams of gallic acid equivalent per gram of dry plant mass), exceeding those of *E. hirsutum* by 16.8%; in flowers, the respective values were 153 ± 3 mg_GAE/g, a difference of 1.3%. Regarding TFC, *E. angustifolium* leaves contained 25 ± 1.4 mg_RE/g (milligrams of rutin equivalent per gram of dry plant mass), which was 20% lower than in *E. hirsutum*, whereas its flowers accumulated 44 ± 1.4 mg_RE/g, representing a 63% higher content compared with *E. hirsutum*. The study may contribute to the selection of the *Epilobium* populations for genetic resource conservation and sustainable utilisation.

## 1. Introduction

An increasing interest in healthy lifestyles and natural products has been observed, particularly in those rich in various active ingredients that are predominantly biosynthesized by plants as secondary metabolites. Polyphenolic compounds, or polyphenols, constitute one of the largest and most important groups of these metabolites [1,2]. This group includes flavonoids, phenolic acids, and tannins—compounds known for their antioxidant, anticancer, antibacterial, cardioprotective, anti-inflammatory, and immune-modulating properties [2].

Among the plants that accumulate phenolic compounds, narrow-leaved willowherb (*Epilobium angustifolium* L., syn. *Chamerion angustifolium* (L.) Holub) is well known, particularly in folk medicine [3]. Willowherbs have long been used in traditional remedies to treat a variety of conditions, ranging from digestive disorders to skin inflammations and other topical applications [4,5,6]. The herbal medicinal product *Epilobii herba* (prepared from the aerial parts of *E. angustifolium* and/or *E. parviflorum*) has been approved by the Committee on Herbal Medicinal Products (HMPC) of the European Medicines Agency and, based on traditional use, is recommended for the treatment of urinary tract and genital disorders [7]. Another congeneric species, hairy willowherb (*Epilobium hirsutum* L.), has also been used for similar purposes in folk medicine, though it is far less popular [8,9]. Traditional uses of *Epilobium* species include decoctions, infusions, poultices, and teas [9] as well as recreational teas commonly consumed in Eastern Europe [10,11]. Currently, teas produced from fermented willowherb leaves are gaining popularity due to their higher polyphenol content and improved aroma and flavor [12,13]. Interestingly, a recent study by Safa et al. (2025) reported that optimized herbal extracts of *Epilobium hirsutum* demonstrated the strongest *in vitro* wound-healing activity, outperforming well-known species such as *Sambucus nigra* and *Lythrum salicaria* [14]. This suggests that *E. hirsutum* holds potential for medicinal applications.

The above-mentioned *Epilobium* species are perennial plants naturally distributed across temperate biomes [15,16] and are also common in Lithuania. *E. angustifolium* is frequently found in recently cleared land, forest clearings, edges of forests, and areas affected by fire, while *E. hirsutum* thrives in fens, marshes, and moist, wet grassland habitats. *E. angustifolium* prefers slightly acidic to near-neutral soil reaction, while *E. hirsutum* is associated with alkaline and eutrophic soils [17]. Taxonomically, the two species are attributed to different sections, with *Epilobium angustifolium* representing *E.* sect. *Chamaenerion* and *E. hirsutum* being part of *E.* sect. *Epilobium* [18]. Recent studies employing spectrophotometric techniques have shown that the leaves and flowers of *E. angustifolium* collected in Ukraine are characterized by high levels of polyphenols and triterpenoids, and that the highest polyphenol content occurs during the late flowering stage [19]. Studies conducted on five *Epilobium* species in Estonia have shown that the highest proportional levels of polyphenols in leaves were found in *E. hirsutum* (19.3 ± 1.5%) [20,21].

Quantitative estimation of polyphenolic compounds in *Epilobium* medicinal raw materials—taking into account growth conditions, phenological stage, and processing method—is essential for assessing their potential as sources of natural products and for supporting their broader use in health maintenance and therapy. Spectrophotometry remains one of the most widely applied methods for estimating these compounds, as it measures light absorption following the reaction of analytes with specific reagents. This method offers notable advantages, including rapidity and ease of use, enabling the determination of both total polyphenol content and specific polyphenolic groups.

The aim of this work was to estimate and compare the total phenolic content (TPC) and total flavonoid content (TFC) in the medicinal raw materials—leaves and flowers—of *Epilobium angustifolium* L. and *Epilobium hirsutum* L. collected from different populations in Lithuania, using simple and cost-effective spectrophotometric methods: the Folin–Ciocalteu assay (for TPC) and the aluminium chloride (AlCl_3_) assay (for TFC). As the Folin–Ciocalteu assay measures overall reducing capacity rather than being phenolic-specific, we regard the resulting TPC values as estimates rather than direct measures of phenolic content. The aluminium chloride (AlCl_3_) assay is likewise not highly specific. Therefore, in this study, we use the term ‘estimation’ when referring to TPC and TFC. We hypothesized that, despite being less extensively studied, *E. hirsutum* may possess phenolic compound levels comparable to those of the better-known *E. angustifolium*, and that both species may exhibit significant within-species variation enabling the selection of the most productive populations for the conservation and use of genetic resources.

## 2. Results

### 2.1. Estimation of Total Phenolic Contents (TPC) in Leaves of Epilobium angustifolium and E. hirsutum

**Figure 1:**
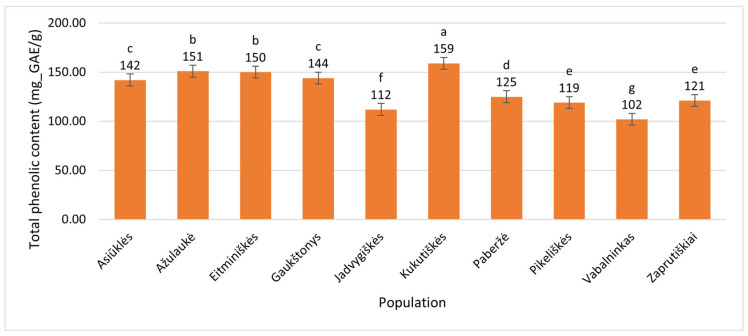
Total phenolic contents (mg_GAE/g of dry plant mass) in leaves of *Epilobium angustifolium* collected from different populations. Population names follow the names of the nearest villages. Error bars indicate standard errors of the mean (N = 3). Different letters above the bars indicate significant differences between populations (*p* ≤ 0.05, Duncan’s Multiple Range Test).

The total phenolic content (TPC) was quantitatively estimated by conversion to gallic acid equivalents (GAE). Among the 10 examined *E. angustifolium* populations, leaf TPC ranged from 102 ± 1 mg_GAE/g (Vabalninkas, Biržai District) to 159 ± 0.3 mg_GAE/g (Kukutiškės, Vilnius District) (Figure 1), corresponding to a 55.9% increase over the minimum average value.

**Figure 2:**
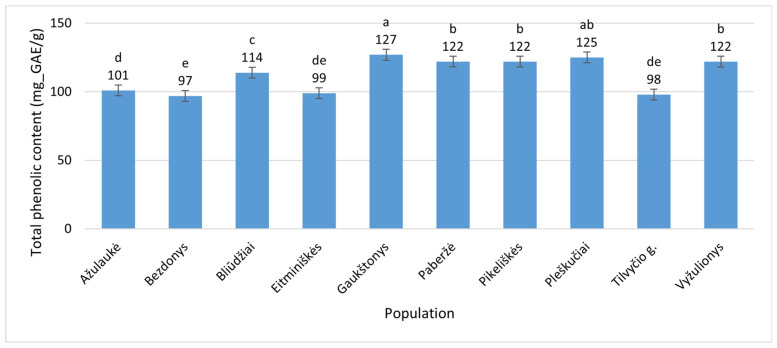
Total phenolic contents (mg_GAE/g) in leaves of *Epilobium hirsutum* collected from different populations. Population names follow the names of the nearest villages. Error bars indicate standard errors of the mean (N = 3). Different letters above the bars indicate significant differences between populations (*p* ≤ 0.05, Duncan’s Multiple Range Test).

Meanwhile, the TPC in *E. hirsutum* leaves varied within a slightly lower and narrower range, from 96 ± 0.36 mg_GAE/g (Bezdonys, Vilnius District) to 127 ± 2.3 mg_GAE/g (Gaukštonys, Vilnius District) (Figure 2), representing a 32.3% difference relative to the minimal average value.

### 2.2. Estimation of Total Phenolic Contents (TPC) in Flowers of Epilobium angustifolium and E. hirsutum

**Figure 3:**
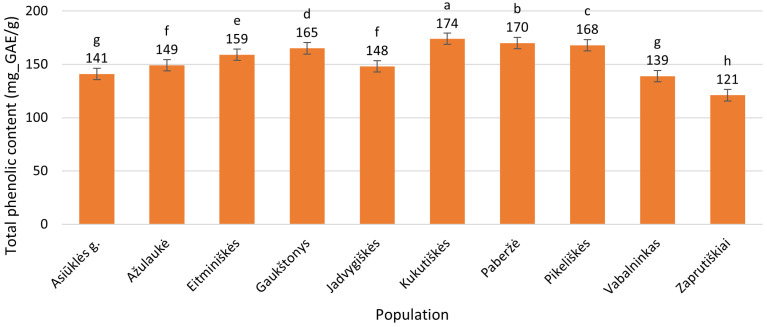
Total phenolic contents (mg_GAE/g) in flowers of *Epilobium angustifolium* collected from different populations. Population names follow the names of the nearest villages. Error bars indicate standard errors of the mean (N = 3). Different letters above the bars indicate significant differences between populations (*p* ≤ 0.05, Duncan’s multiple range test).

Flowers of *Epilobium angustifolium* contained between 121 ± 0.37 mg_GAE/g (Zaprutiškiai, Panevėžys District) and 174 ± 1.5 mg_GAE/g (Kukutiškės, Vilnius District) (Figure 3), showing a 43.8% variation relative to the minimal average value.

**Figure 4:**
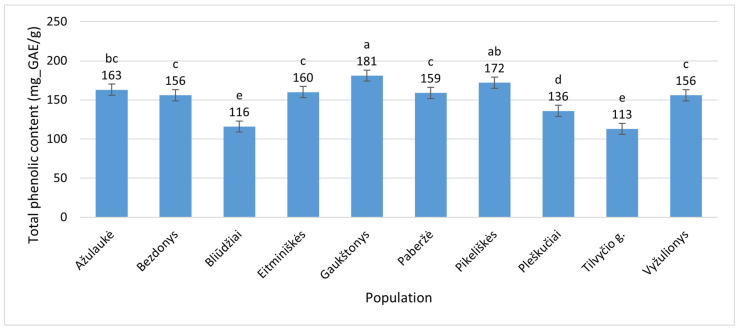
Total phenolic contents (mg_GAE/g) in flowers of *Epilobium hirsutum* collected from different populations. Population names follow the names of the nearest villages. Error bars indicate standard errors of the mean (N = 3). Different letters above the bars indicate significant differences between populations (*p* ≤ 0.05, Duncan’s multiple range test).

The flowers of *E. hirsutum* contained between 113 ± 0.78 mg_GAE/g (Tilvyčio g., Panevėžys District) and 181 ± 1.3 mg_GAE/g (Gaukštonys, Vilnius District) (Figure 4), indicating a relatively high variation of 60.2% relative to the minimum average value.

### 2.3. Comparison of Total Phenolic Contents (TPC) in Leaves and Flowers of Epilobium angustifolium and E. hirsutum

To find out whether there were any consistent patterns among populations distinguished by TPC, we compiled a summary table of the estimated minimum and maximum TPC values (Table 1).

As shown in Table 1, the Kukutiškės population was distinguished by its higher TPC in both the leaves and flowers of *E. angustifolium*, with differences of 55.9% and 43.8%, respectively. Meanwhile, the Gaukštonys population showed the highest TPC in the leaves and flowers of *E. hirsutum*, with differences amounting to 30.9% and 60.2%, respectively.

**Figure 5:**
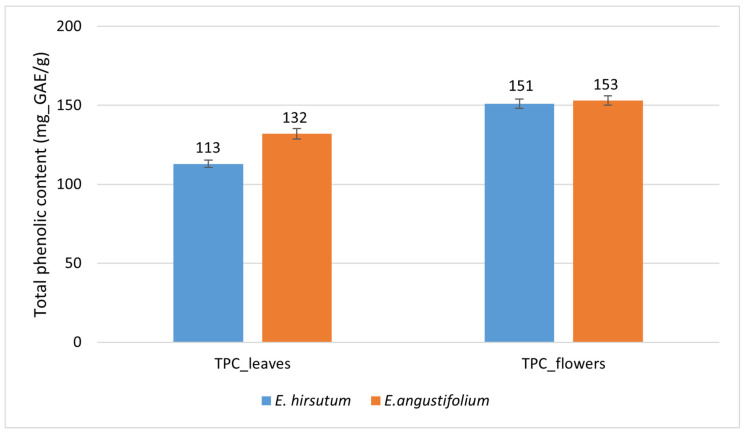
Between-species comparison of total phenolic contents (mg_GAE/g) in leaves (significant difference, *p* < 0.001) and flowers (non-significant difference, *p* = 0.681) of *Epilobium angustifolium* and *E. hirsutum*, pooled from multiple populations. N = 10; error bars represent standard errors (SE).

We also estimated the TPC at the species level. The average content of phenolic compounds (mg_GAE/g) in *E. angustifolium* leaves was 132 ± 3.4, while in *E. hirsutum* leaves it was 113 ± 2.2 (Figure 5), representing a difference of 19 mg_GAE/g, or 16.8% relative to *E. hirsutum*. An independent samples *t*-test showed that this difference was statistically significant (*p* < 0.001), and the calculated effect size (Cohen’s d = 1.26) indicated a large species effect. These results suggest that *E. angustifolium* is superior to *E. hirsutum* in terms of TPC in leaves.

Meanwhile, when comparing the estimates of phenolic compounds in the flowers, no statistically significant difference was established between the species. The amount of phenolic compounds in the flowers of *E. angustifolium* was 153 ± 3.0, and in the flowers of *E. hirsutum*, 151 ± 3.0 mg_GAE/g (Figure 5), making the difference of only 1.3%. The *t*-test showed that the difference between means (2 mg_GAE/g) was statistically non-significant (*p* = 0.681), and the effect size index (Cohen’s d = 0.11) showed a very small effect of the plant species. Therefore, both species can be considered comparable based on the TPC of their flowers. Furthermore, the univariate ANOVA indicated a very large species effect on leaf TPC (partial η^2^ = 0.970, *p* < 0.001). However, the population effect was even larger, as reflected by a partial η^2^ of 0.991 (*p* < 0.001). Meanwhile, the effect of species on TPC in flowers accounted for 8.7% of the variance (partial η^2^ = 0.087). However, the effect did not reach statistical significance (*p* = 0.057). Population had a very large effect on TPC in flowers (partial η^2^ = 0.969, *p* < 0.001), indicating that approximately 97% of the variance is attributable to population differences.

### 2.4. Estimation of Total Flavonoid Contents (TFC) in Leaves of Epilobium angustifolium and E. hirsutum

**Figure 6:**
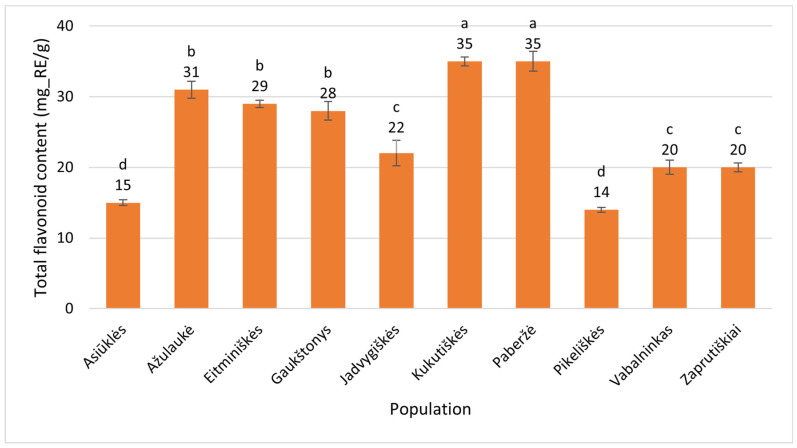
Total flavonoid contents (mg_RE/g of dry plant mass) in leaves of *Epilobium angustifolium* collected from different populations. Population names follow the names of the nearest villages. Error bars indicate standard errors of the mean (N = 3). Different letters above the bars indicate significant differences between populations (*p* ≤ 0.05, Duncan’s Multiple Range Test).

The TFC in *Epilobium angustifolium* leaves ranged from 14 ± 0.35 mg_RE/g (Pikeliškės, Vilnius District) to 35 ± 0.62 mg_RE/g (Kukutiškės, Vilnius District). The Paberžė population also showed a high TFC in *E. angustifolium* leaves, although with slightly greater variation (35 ± 1.5 mg_RE/g) (Figure 6). Thus, the highest value was 2.5 times greater than the lowest, and this difference was statistically significant (*p* < 0.001).

**Figure 7:**
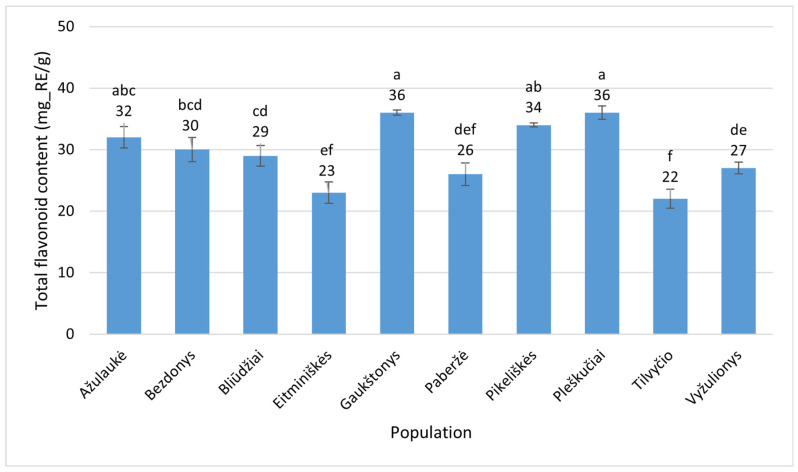
Total flavonoid contents (mg_RE/g) in leaves of *Epilobium hirsutum* collected from different populations. Population names follow the names of the nearest villages. Error bars indicate standard errors of the mean (N = 3). Different letters above the bars indicate significant differences between populations (*p* ≤ 0.05, Duncan’s multiple range test).

In *E. hirsutum* leaves, the TFC ranged from 22 ± 1.5 mg_RE/g (Tilvyčio g., Panevėžys District) to 36 ± 0.42 mg_RE/g (Gaukštonys, Vilnius District). The Pleškučiai population also showed a high TFC in *E. hirsutum* leaves, although with slightly greater variation (36 ± 1.1 mg_RE/g) (Figure 7). Thus, the highest value was 1.6 times greater than the lowest, and this difference was statistically significant (*p* < 0.001).

### 2.5. Estimation of Total Flavonoid Contents (TFC) in Flowers of Epilobium angustifolium and E. hirsutum

**Figure 8:**
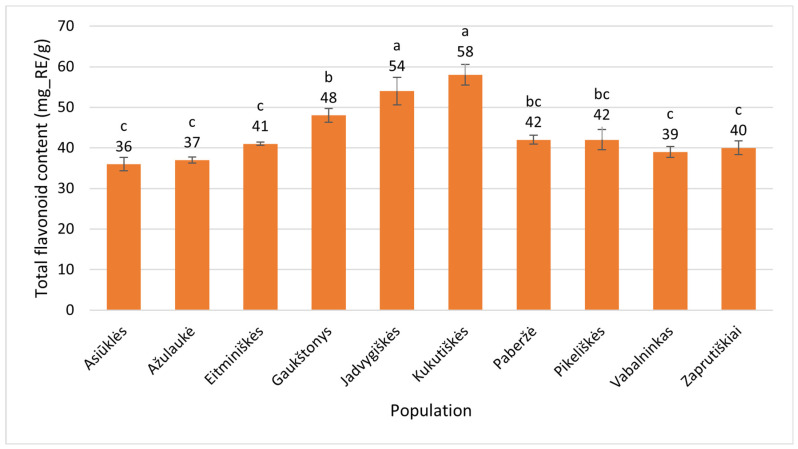
Total flavonoid contents (mg_RE/g) in flowers of *Epilobium angustifolium* collected from different populations. Population names follow the names of the nearest villages. Error bars indicate standard errors of the mean (N = 3). Different letters above the bars indicate significant differences between populations (*p* ≤ 0.05, Duncan’s multiple range test).

The estimates of total flavonoid content (mg_RE/g) in the flowers of *E. angustifolium* ranged from 36 ± 1.6 (Asiūklės g., Vilnius District) to 58 ± 2.6 (Kukutiškės, Vilnius District) (Figure 8). The latter value exceeded the former by 1.6 times. The differences observed were statistically significant (*p* < 0.001).

**Figure 9:**
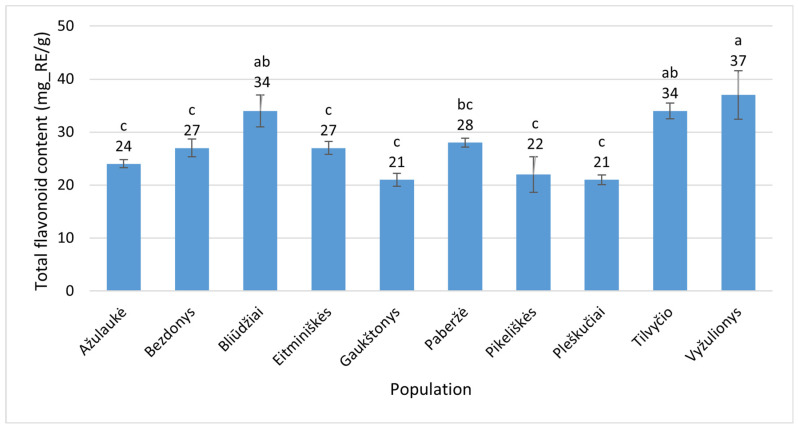
Total flavonoid contents (mg_RE/g) in flowers of *Epilobium hirsutum* collected from different populations. Population names follow the names of the nearest villages. Error bars indicate standard errors of the mean (N = 3). Different letters above the bars indicate significant differences between populations (*p* ≤ 0.05, Duncan’s multiple range test).

The estimates of TFC (mg_RE/g) in the flowers of *Epilobium hirsutum* ranged from 21 ± 0.91 and 21 ± 1.2 (Pleškučiai, Klaipėda District, and Gaukštonys, Vilnius District, respectively) to 37 ± 4.5 (Vyžulionys, Vilnius District) (Figure 9). The latter value exceeded the former ones by 1.8 times. The differences observed were statistically significant (*p* < 0.001).

### 2.6. Comparison of Total Flavonoid Contents (TFC) in Leaves and Flowers of Epilobium angustifolium and E. hirsutum

To find out whether there were any consistent patterns among populations distinguished by the TFC, we compiled a summary table of the estimated minimum and maximum TFC values (Table 2).

As shown in Table 2, the Kukutiškės population exhibited the highest TFC in both the leaves and flowers of *E. angustifolium*, consistent with the TPC patterns observed in Table 1. In contrast, no such consistency was found for *E. hirsutum*: the Gaukštonys population showed the highest estimates of TFC in leaves (aligning with the TPC data; see Table 1), whereas the Vyžulionys population displayed the highest TFC in flowers, which did not correspond to the TPC distribution (see Table 1). The percentage differences between the estimated minimum and maximum TFC values were generally higher (61.1–150%, observed in *E. angustifolium*, Table 2) than those of TPC distribution (30.9–60.2%, observed in *E. hirsutum*, Table 1).

**Figure 10:**
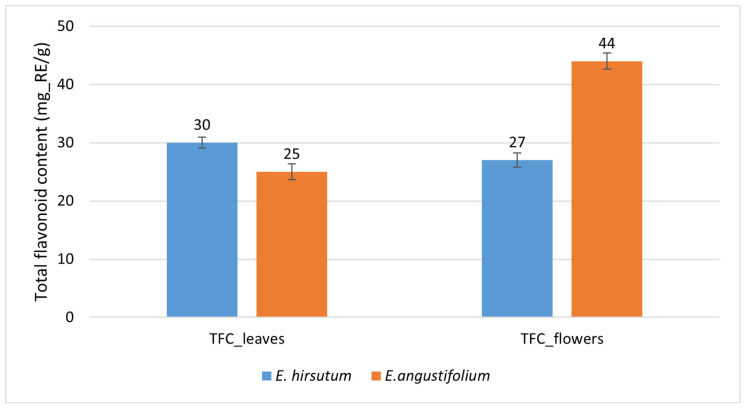
Between-species comparison of total flavonoid contents (mg_RE/g) in leaves (significant difference, *p* = 0.008) and flowers (significant difference, *p* < 0.001) of *Epilobium angustifolium* and *E. hirsutum*, pooled from multiple populations. N = 10; error bars represent standard errors (SE).

We also estimated the TFC at the species level. The average TFC (mg_RE/g) in *E. angustifolium* leaves was 25 ± 1.4, while in *E. hirsutum* leaves it was 30 ± 0.96 (Figure 10), indicating a statistically significant difference of 5.0 mg_RE/g (16.7% relative to *E. hirsutum*; *p* = 0.008). The effect size (Cohen’s d = 0.71) indicated a medium species effect. In contrast, the pattern was reversed in the flowers: *E. angustifolium* accumulated significantly more flavonoids than *E. hirsutum* (44 ± 1.4 vs. 27 ± 1.2 mg_RE/g, respectively), with a difference of 17 mg_RE/g (63.0% relative to *E. hirsutum*; *p* < 0.001). The corresponding effect size (Cohen’s d = 2.27) indicated a large species effect (Figure 10).

This indicates that *E. angustifolium* is superior to *E. hirsutum* in terms of TFC in flowers, whereas *E. hirsutum* likely exceeds its congener in TFC in leaves. Furthermore, the univariate ANOVA indicated that species had a very large effect on TFC in leaves (partial η^2^ = 0.624, *p* < 0.001), indicating that approximately 62% of the variance is attributable to species differences. However, population had an extremely large effect on leaf TFC (partial η^2^ = 0.931, *p* < 0.001), indicating that about 93% of the variance is attributable to population differences. Species also had an extremely large effect on TFC in flowers (partial η^2^ = 0.882, *p* < 0.001), indicating that about 88% of the variance is attributable to species differences. Population had a very strong effect on TFC in flowers (partial η^2^ = 0.924, *p* < 0.001), showing that about 92% of the variance is explained by population differences.

### 2.7. The Effects of Location and Species on TPC and TFC

Since the two studied species were sampled at four common locations (Ažulaukė, Eitminiškės, Gaukštonys, and Pikeliškės (seeSection 4), we evaluated the effects of location and species on total phenolic and total flavonoid contents (Table 3).

As shown in Table 3, the partial eta squared (η^2^_p_) values indicated very large effects of species (η^2^_p_ = 0.986), location (η^2^_p_ = 0.912), and their interaction (η^2^_p_ = 0.978) on TPC in leaves. Very similar effect sizes were obtained for TPC in flowers. These results demonstrate that species identity explains almost all variation in leaf phenolic content, while site-specific environmental factors and species-specific responses to sites also contribute substantially. The effect of location was strongest for two variables: TPC in flowers (η^2^_p_ = 0.983) and TFC in leaves (η^2^_p_ = 0.825).

## 3. Discussion and Conclusions

The total phenolic and total flavonoid contents estimated in the present study cannot be directly compared with those reported by other researchers, because TPC and TFC values are strongly affected by sample type and analytical methodology. Many studies use different plant materials (e.g., aerial parts or whole herbs rather than separated leaves and flowers), different phenological stages, and different extraction solvents and protocols (such as ethanol vs. methanol, varying solvent concentrations, or maceration vs. sonication), all of which can significantly affect the measured values. For example, Umińska et al. (2025) [22] found 193.35 mg/g of phenolics in the aerial parts of *E. angustifolium*, a value that exceeds the maximum TPC estimate obtained in our *E. angustifolium* flowers by 11.2%. In contrast, their reported phenolic content for *E. hirsutum* (181.58 mg/g) closely matches our maximum estimate for this species (181.39 mg/g; Gaukštonys population). It should be noted, however, that these data were obtained using a different analytical technique (HPLC–PDA) and cannot be compared directly.

Düüna et al. (2023) [23] reported high polyphenol concentrations in *E. angustifolium* based on colorimetric assays, reaching up to 157.6 ± 8.1 mg_GAE/g, which is very close to the maximum TPC we estimated in *E. angustifolium* leaves (158.74 mg_GAE/g; Kukutiškės population). Meanwhile, data from Turkey indicate that the TPC of the aerial parts of *E. hirsutum* may reach 254.55 ± 0.72 mg_GAE/g [24], more than twice the estimates obtained in our study. Also, their reported TFC value (87.66 ± 0.38 mg_RE/g) was more than twice as high as our corresponding data (37 ± 4.5 mg_RE/g). These discrepancies may also be attributed to the differences in climatic conditions—which are known to strongly influence the accumulation of phenolic compounds—as well as differences in genotypes.

It is known that the Folin–Ciocalteu (F–C) method measures total reducing capacity or antioxidant activity, meaning that non-phenolic compounds capable of reducing the F–C reagent may contribute to an overestimation of total phenolic content [25] in addition to other biochemical factors that fall outside the scope of the present study. One major non-phenolic interferent is ascorbic acid (vitamin C), which is present in many plant species, including willowherbs. For example, it has been reported that *E. angustifolium* leaves accumulate 6.78 ± 0.11 mg_AA/g (milligram of gallic acid equivalent per gram of dry plant mass) of vitamin C [26]. Meanwhile, no quantitative data is available on the vitamin C content of *E. hirsutum*, apart from general statements indicating that the species is rich in this compound. If we use the above-reported vitamin C value (6.78 mg_AA/g) as a reference, and apply the relative response of ascorbic acid to the F–C reagent reported by Everette et al. (2010) [27], where ascorbic acid exhibits 0.662 of the gallic acid signal on a mass basis, then ascorbic acid would account for approximately 3% of the average TPC in *E. angustifolium* leaves determined in the present study (132 mg_GAE/g; Figure 5). Thus, the potential overestimation of TPC in our study is likely to be of that magnitude. Nevertheless, the colorimetric assays used in this study should be valued as effective tools due to their simplicity, rapidity, and cost-effectiveness—features that are particularly valuable for screening of large germplasm collections and resource-limited settings.

In support of our hypothesis, the present study showed that the less-studied species *Epilobium hirsutum* could be considered a substitute for *E. angustifolium* or serve as an additional source of phenolic compounds. Two findings support this conclusion: both species exhibit similar levels of total phenolic contents in their flowers, and *E. hirsutum* is likely to exceed its congener in total flavonoid content in the leaves. Our study results also indicate that growth conditions have a greater impact on TPC and TFC levels than the species itself. Furthermore, because these species often occupy different habitats, this may improve the resource supply, making it more evenly distributed across the territory. Therefore, efforts to standardize *E. hirsutum* leaves through a pharmacopoeial monograph are likely already underway. Perhaps the first such effort was recently undertaken by a group of researchers from Ukraine and Lithuania [28], who concluded that total phenolic content in the leaves could be proposed as one of the evaluation criteria for *E. hirsutum* medicinal raw material.

A significant outcome of the present study is the identification of candidate populations of the examined *Epilobium* species that may be suitable for in situ and/or ex situ conservation and for use as genetic resources. In particular, the Kukutiškės population of *E. angustifolium* and the Gaukštonys population of *E. hirsutum* emerge as primary candidates, as they stand out for their estimated total phenolic content (Table 1) and total flavonoid content (Table 2). This finding demonstrates that the spectrophotometric methods applied in this study can serve effectively as screening tools for the characterization and preselection of the populations. However, to enable the final selection of the most promising populations, particularly when detailed, individual molecular profiling is required, more precise analytical techniques—such as high-performance liquid chromatography—should be applied. Furthermore, supplementary information, such as site effects, multi-year sampling, common-garden experiments, and genetic analyses, is essential to substantiate the selection of populations for long-term conservation.

Future research should include other *Epilobium* species native to the region, among which *Epilobium montanum* L. and *E. obscurum* Schreb. remain some of the least studied. The determination of tannins—particularly oenothein B, the major active compound in *Epilobium* species, and a key marker for the standardization of *Epilobium* raw material [8,29]— should be considered a priority for future studies.

## 4. Materials and Methods

### 4.1. Sampling of Plant Materials

Sampling of flowering tops (upper one-third of the stem) of *Epilobium angustifolium* L. and *Epilobium hirsutum* L. (Onagraceae), up to 30–40 ones per population, was carried out during the first week of July 2024. Ten populations were sampled for each species, mostly from eastern and north-eastern parts of Lithuania (Table 4).

The collected plant samples were dried in a well-ventilated room, protected from direct sunlight, in a single layer on a solid surface lined with brown packing paper. During the initial week of drying, the plant material was stirred daily to promote uniform drying; thereafter, stirring was performed less frequently. The samples were left to air-dry for two weeks at an ambient temperature of 19–21 °C. The final water content of the dried plant samples ranged from 7.7% to 9.9%, which is consistent with standard values for air-dried medicinal herbs. The dried plant material was divided into flowers and leaves by picking single leaves and whole inflorescences from each plant sample and stored in paper bags at a temperature not exceeding 25 °C, in a dry place, protected from direct sunlight for about four months before analysis. The remaining plant material (stems and non-selected parts) was retained as spare material and stored for potential further analyses.

### 4.2. Reagents and Laboratory Equipment

Gallic acid, Folin–Ciocalteau reagent, sodium carbonate (Na_2_CO_3_) and methanol were used to determine the total phenolic contents in plant raw materials by the Folin–Ciocalteau colorimetric method. Hexamethylenetetramine, aluminum chloride (AlCl_3_), rutin, methanol and acetic acid were used to determine the total flavonoid contents in plant raw materials by the spectrophotometric method. All chemicals used in this study were of analytical (laboratory) grade; solvents were of analytical or HPLC grade, and reagents were as follows: Folin & Ciocalteu’s phenol reagent (47641-100, 2M, Sigma-Aldrich, St. Louis, MO, USA), gallic acid (≥98% purity, ACS, anhydrous, CAS: 149-91-7, Carl ROTH GmbH + Co. KG, Karlsruhe, Germany), and rutin trihydrate (≥95%, Ph. Eur., Art. No. 5154.1, Carl ROTH), aluminium chloride (≥99%, anhydrous, sublimated, Carl ROTH), hexamethylene tetramine (ROTIPURAN^®^ ≥99%, p.a., ACS, Art. No. 4366.1, Carl ROTH), sodium carbonate (≥99%, anhydrous, Art. No. 8563.1, Carl Roth), acetic acid (100%, Ph. Eur., extra pure, Art. No. 6755.1, Carl ROTH), methanol (Puriss. p.a., ACS, Ph. Eur., ≥99.8% (GC), Honeywell Riedel-de Haën, Seelze, Germany). The purified water used in all experiments met laboratory-grade specifications: specific conductivity ≤1.0 µS/cm (corresponding to ≥1 MΩ·cm resistivity), and total dissolved solids <1 mg/L.

A knife mill (Retsch Grindomix 200; Retsch GmbH, Haan, Germany) was used to grind the plant material to less than 0.5 mm of grain size (as estimated by passing the material through a corresponding mesh sieve), and an ultrasonic bath (Argo Lab DU-32, ultrasonic power 120 W, Argo Lab, Italy) and a centrifuge (Eppendorf 5430R; Eppendorf SE, Hamburg, Germany) were used to extract the samples. A C-7200S UV-Vis spectrophotometer (PEAK Instruments, Houston, TX, USA) was used to determine the total phenolic contents and total flavonoid contents in the leaves and flowers of *Epilobium angustifolium* L. and *Epilobium hirsutum* L.

### 4.3. Preparation of Plant Extracts

Extracts were prepared from 0.05 g of ground leaves and flowers by adding 20 mL of 70% (*v*/*v*) methanol and extracting the mixture in an ultrasonic bath (Argo Lab DU-32) for 20 min at 40 kHz, with an ultrasonic power of 120 W and heating power of 100 W. The water bath temperature was maintained at 40 °C. No additional mechanical agitation was applied beyond the ultrasound-induced cavitation. The obtained extracts were centrifuged for 10 min at 4 °C at a relative centrifugal force of 4226× *g* in a centrifuge (Eppendorf 5430R). The obtained clear extracts were transferred to 25 mL volumetric flasks and diluted to volume with 70% (*v*/*v*) methanol. In this way, 30 leaf extracts (N = 3 pooled-material extracts per population) and 30 flower extracts (N = 3 pooled-material extracts per population) were prepared for each species. All extracts were stored under refrigerated conditions (4 °C) and were analyzed on the same day they were prepared. Completing the full set of analyses for all samples required approximately six weeks in total.

### 4.4. Estimation of Total Phenolic Content

The following working reagents were prepared first: 70% methanol solution, Folin–Ciocalteu (F–C) reagent solution, and sodium carbonate (Na_2_CO_3_) solution. A 1:10 dilution of F–C was made: 10 mL of the reagent was added to a dark glass bottle, followed by 90 mL of distilled water. The solution was mixed gently and then stored at 4 °C, protected from light. The solvent used for preparing the sodium carbonate solution was distilled water, and the concentration of the solution was 20% (200 g/L). A stock solution of gallic acid with a concentration of 5 g/L was prepared by using methanol (70% *v*/*v*) as a solvent, from which standard solutions were further prepared for the calibration curve. For analysis, 0.1 mL of the prepared plant material extract, 7 mL of distilled water, 0.5 mL of Folin–Ciocalteau reagent solution were taken into test tubes, the test tube was gently rotated, and incubated for 8 min at room temperature (approximately 21–23 °C). Then, 1.5 mL Na_2_CO_3_ solution and 0.9 mL distilled water were added to the same test tube to bring the total reaction volume to 7.9 mL. The test tube containing the resulting mixture was tightly closed, then shaken thoroughly while being inverted several times, and left to stand for 15 min. at room temperature (21–23 °C), in a dark place. After the specified time, the absorbance of the solution was measured with a spectrophotometer (C-7200S UV–Vis) at a light wavelength of 765 nm. All measurements were repeated 3 times. The reference solution was prepared in the same way as the test solution, only instead of the plant extract, 0.1 mL of 70% (*v*/*v*) methanol was added.

Standard gallic acid solutions were prepared in the same way as test solutions, only instead of 0.1 mL of plant extract, 0.1 mL of gallic acid solutions of known concentrations was taken. From this, five concentrations of gallic acid solutions were prepared: 0.5; 1.0; 1.5; 2.5; 5.0 mg/mL.

**Figure 11:**
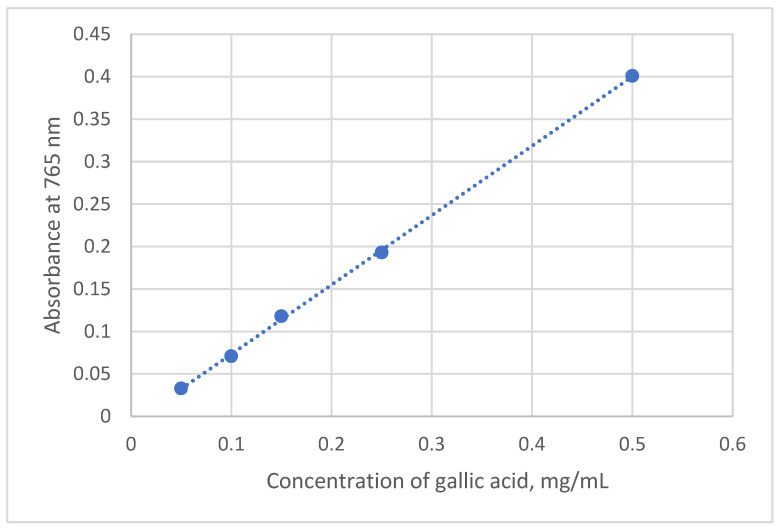
Gallic acid calibration graph for the determination of total phenolic contents.

The obtained data were evaluated according to the linear regression equation of the gallic acid calibration graph (Figure 11):

y = 0.8176x − 0.0085, R^2^ = 0.9997,

where y—absorbance value; x—total phenolic content, expressed as GAE (gallic acid equivalent), mg/mL; R—coefficient of determination.

The total phenolic content in a plant material, expressed as mg GAE/mL, was calculated according to the formula:

C = c × V/m (mg/g),

where C—the total phenolic content in the plant material, expressed as gallic acid equivalent; c—the total phenolic content in the extract of the plant material, expressed as gallic acid equivalent (mg/mL); V—the volume of the extract of the plant material (ml); m—the mass of the ground plant material taken for extraction (g).

### 4.5. Estimation of Total Flavonoid Content

The following working reagents were prepared: 70% (*v*/*v*) methanol solution, standard rutin solution (0.5 mg/mL) (using 70% (*v*/*v*) methanol as the solvent), 10% (*w*/*v*) aluminum chloride (AlCl_3_) solution, 5% (*w*/*v*) hexamethylenetetramine solution, 30% (*v*/*v*) acetic acid solution.

Two rutin solutions were prepared—the rutin reference solution and rutin test solution:

(1) The rutin reference solution was made of 0.1 mL of the prepared standard rutin solution, 1 mL of 100% methanol, 0.05 mL of 30% acetic acid solution and 1.35 mL of distilled water (the nominal volumetric additions were used without correction for methanol–water volume contraction);

(2) The rutin test solution was made of 0.1 mL of the prepared standard rutin solution, 1 mL of 100% methanol, 0.05 mL of 30% acetic acid solution, 0.15 mL of 10% aluminum chloride (AlCl_3_) solution, 0.2 mL of 5% hexamethylenetetramine solution and 1 mL distilled water (the volumes were used as nominal additive volumes without correction for methanol–water contraction); everything was mixed in a test tube, covered with parafilm, and left to incubate at room temperature (21–23 °C) for 20 min.

The optical density of the rutin test solution was measured in a 1 cm cuvette relative to the rutin reference solution, using a wavelength of 407 nm, with the spectrophotometer C-7200S UV–Vis.

The reference and test solutions of the herbal extract were prepared in the same way as the rutin solutions, only instead of 0.1 mL of the standard rutin solution, 0.1 mL of the prepared herbal extract solution was taken. Then, the optical density of the herbal extract test solution was measured in a 1 cm cuvette relative to the herbal extract reference solution, using a wavelength of 407 nm.

The total flavonoid content in a plant material, converted to rutin, is calculated according to the formula:

X = (m_r_ × V_pe_ × D_pe_ × 100)/(m_pe_ × D_r_ × V_r_),

where X—total flavonoid content in the plant material, converted to rutin (%);

m_r_—mass of rutin used to prepare the standard rutin solution (g);

V_pe_—volume of plant extract (mL);

D_pe_—optical density of the test solution of plant extract;

m_pe_—mass of the ground plant material taken for extraction (g);

D_r_—optical density of the standard rutin test solution;

V_r_—volume of the standard rutin solution (mL).

### 4.6. Statistical Data Analysis and Presentation

Data analysis was performed using Microsoft Excel 365 (Microsoft Corporation, Redmond, WA, USA) and IBM SPSS Statistics (Version 30) (IBM Corp., Armonk, NY, USA) software. Statistically significant data reliability was considered when *p* < 0.05. For statistical comparisons between two plant species and between two plant parts, an independent two-sample *t*-test was used. For comparisons of a larger number of data groups, one-way ANOVA was used, followed by Duncan’s new multiple range test. Cohen’s d index was used to determine the effect size. Numerical data were presented according to the concept of significant digits [30,31].

## Figures and Tables

**Table 1:** Summary comparison of *Epilobium angustifolium* and *E. hirsutum* populations by minimal and maximal total phenolic contents (TPC) (M ± SE, mg_GAE/g).

Species	Plant Part	TPC Min (Population)	TPC Max (Population)	Difference	% Diff of Min
*E. angustifolium*	Leaves	102 ± 1 (Vabalninkas)	159 ± 0.3 (Kukutiškės)	57	55.9
	Flowers	121 ± 0.37 (Zaprutiškiai)	174 ± 1.5 (Kukutiškės)	53	43.8
*E. hirsutum*	Leaves	97 ± 0.36 (Bezdonys)	127 ± 2.3 (Gaukštonys)	30	30.9
	Flowers	113 ± 0.78 (Tilvyčio g.)	181 ± 1.3 (Gaukštonys)	68	60.2

**Table 2:** Summary comparison of *Epilobium angustifolium* and *E. hirsutum* populations by minimal and maximal total flavonoid contents (TFC) (M ± SE, mg_RE/g).

Species	Plant Part	TFC Min (Population)	TFC Max (Population)	Difference	% Diff of Min
*E. angustifolium*	Leaves	14 ± 0.35 (Pikeliškės)	35 ± 0.62 (Kukutiškės)	21	150
	Flowers	36 ± 1.6 (Asiūklės g.)	58 ± 2.6 (Kukutiškės)	22	61.1
*E. hirsutum*	Leaves	22 ± 1.5 (Tilvyčio g.)	36 ± 0.42 (Gaukštonys)	14	63.6
	Flowers	21 ± 0.91 (Pleškučiai)	37 ± 4.5 (Vyžulionys)	16	76.2

**Table 3:** Two-way ANOVA results of evaluation of species and location effects on total phenolic contents (TPC) and total flavonoid contents (TFC) based on four common locations of *Epilobium angustifolium* and *E. hirsutum*.

Variable	Source of Effect	df	F	*p*	Effect Size η^2^_p_
TPC (leaves)	Species	1	1107.08	<0.001	0.986
	Location	3	55.60	<0.001	0.912
	Species × Location	3	238.12	<0.001	0.978
TPC (flowers)	Species	1	354.97	<0.001	0.957
	Location	3	305.67	<0.001	0.983
	Species × Location	3	69.89	<0.001	0.929
TFC (leaves)	Species	1	51.50	<0.001	0.763
	Location	3	25.19	<0.001	0.825
	Species × Location	3	48.40	<0.001	0.901
TFC (flowers)	Species	1	221.36	<0.001	0.933
	Location	3	2.10	0.141	0.282
	Species × Location	3	6.77	0.004	0.559

**Table 4:** Sampling data of *Epilobium angustifolium* and *E. hirsutum* plant materials.

Species	Coordinates, WGS-84	Population Designation	Municipality
*Epilobium angustifolium*	54.838665, 25.266951	Asiūklės g. Riešė	Vilnius District
*Epilobium angustifolium*	54.866143, 25.394302	Ažulaukė	Vilnius District
*Epilobium angustifolium*	54.923083, 25.368639	Eitminiškės	Vilnius District
*Epilobium angustifolium*	54.869556, 25.434028	Gaukštonys	Vilnius District
*Epilobium angustifolium*	54.820778, 25.269056	Jadvygiškės	Vilnius District
*Epilobium angustifolium*	54.833476, 25.505398	Kukutiškės	Vilnius District
*Epilobium angustifolium*	54.929636, 25.235918	Paberžė	Vilnius District
*Epilobium angustifolium*	54.876722, 25.264250	Pikeliškės	Vilnius District
*Epilobium angustifolium*	55.987615, 24.751427	Vabalninkas	Biržai District
*Epilobium angustifolium*	55.963804, 24.723653	Zaprutiškiai	Biržai District
*Epilobium hirsutum*	54.863994, 25.395853	Ažulaukė	Vilnius District
*Epilobium hirsutum*	54.799719, 25.511587	Bezdonys	Vilnius District
*Epilobium hirsutum*	55.767244, 24.299905	Bliūdžiai	Panevėžys District
*Epilobium hirsutum*	54.923006, 25.368544	Eitminiškės	Vilnius District
*Epilobium hirsutum*	54.869535, 25.434008	Gaukštonys	Vilnius District
*Epilobium hirsutum*	54.949914, 25.236939	Paberžė	Vilnius District
*Epilobium hirsutum*	54.876650, 25.264248	Pikeliškės	Vilnius District
*Epilobium hirsutum*	55.537418, 21.312939	Pleškučiai	Klaipėda District
*Epilobium hirsutum*	55.698290, 24.287290	Tilvyčio g. Molainiai	Panevėžys District
*Epilobium hirsutum*	54.940305, 25.286198	Vyžulionys	Vilnius District

## Data Availability

The original contributions presented in this study are included in the article. Further inquiries can be directed to the corresponding author.

## Abbreviations

The following abbreviations are used in this manuscript:

TPC	Total phenolic content
TFC	Total flavonoid content
GAE	Gallic acid equivalent
RE	Rutin equivalent
